# NLRP3 inflammasome activation promotes inflammation-induced carcinogenesis in head and neck squamous cell carcinoma

**DOI:** 10.1186/s13046-017-0589-y

**Published:** 2017-09-02

**Authors:** Cong-Fa Huang, Lei Chen, Yi-Cun Li, Lei Wu, Guang-Tao Yu, Wen-Feng Zhang, Zhi-Jun Sun

**Affiliations:** 1 The State Key Laboratory Breeding Base of Basic Science of Stomatology (Hubei-MOST) & Key Laboratory of Oral Biomedicine Ministry of Education, School & Hospital of Stomatology, Wuhan University, Wuhan, China; 2 Department of Oral Maxillofacial-Head Neck Oncology, School and Hospital of Stomatology, Wuhan University, Wuhan, China

**Keywords:** NLRP3, Inflammasome, SCCHN, Cancer stem cells

## Abstract

**Background:**

NLRP3 inflammasome acts as a danger signal sensor that triggers and coordinates the inflammatory response. However, the roles of NLRP3 inflammasome in the tumorigenesis and development of cancer stem cells (CSCs) of squamous cell carcinoma of the head and neck (SCCHN) remain ambiguous.

**Methods:**

In our study, tissue microarrays, ELISA, sphere-forming assay, colony formation assay and Western blot analysis were performed to evaluate the effect of NLRP3 inflammasome on the development of CSCs in human SCCHN tissue specimen, cell lines, and transgenic mouse SCCHN model.

**Results:**

The components of NLRP3 inflammasome, namely, NLRP3, ASC, Caspase-1, and IL-18 were correlated with CSCs markers BMI1, ALDH1 and CD44 in human SCCHN specimens. Moreover, NLRP3, Caspase-1, IL-1β, and IL-18 were highly expressed in SCCHN cell lines. NLRP3 inflammasome activated by LPS and ATP promoted sphere-forming and colony formation capacities along with an upregulation of BMI1, ALDH1 and CD44. In addition, NLRP3 inflammasome blockade by NLRP3 inhibitor MCC950 reduced sphere and colony number, also decreased the expression of BMI1, ALDH1 and CD44 in SCCHN cell lines. Expression of NLRP3, ASC, Caspase-1, IL-1β, IL-18, BMI1, ALDH1 and CD44 was upregulated in *Tgfbr1*/*Pten* 2cKO mouse SCCHN model, and NLRP3 inflammasome expression was closely related to those CSCs makers in mice SCCHN. However, MCC950 treatment reduced the expression of NLRP3 inflammasome, CSCs markers BMI1, ALDH1 and CD44 in *Tgfbr1*/*Pten* 2cKO mice SCCHN. In addition, blockade of NLRP3 inflammasome can also delayed the tumor-burdened speed in SCCHN mice.

**Conclusions:**

Our study demonstrates that NLRP3 inflammasome was upregulated and associated with the carcinogenesis and CSCs self-renewal activation in SCCHN. NLRP3 inflammasome can be a potential target in the development of novel approaches for head and neck squamous cell carcinoma therapy.

**Electronic supplementary material:**

The online version of this article (10.1186/s13046-017-0589-y) contains supplementary material, which is available to authorized users.

## Background

Squamous cell carcinoma of head and neck (SCCHN) is one of the major causes of mortality and ranks as the sixth most frequent human cancer globally [1]. The incidence of SCCHN in China has been increasing rapidly according to cancer statistics for 2015 [2]. Tobacco, excessive alcohol consumption, and betel quid and derivative consumption are the major risk factors for SCCHN development. The 5-year survival rate of patients with SCCHN has not significantly improved for the past three decades [3]. However, the cellular and molecular mechanisms that contribute to the initiation and progression of SCCHN remain ambiguous.

Inflammation is one of the hallmarks of cancer [4]. However, acute inflammation protects against infectious pathogens. By contrast, chronic inflammation is associated with DNA and tissue damage, which includes genetic and epigenetic changes leading to cancer, such as SCCHN. Inflammasome, an intracellular multi-protein complex, switches on the inflammatory response of tissues to various stimuli, including microbe-derived products, environmental factors, and endogenous molecules [5]. The NLRP3 inflammasome, which is currently the most characterized inflammasome, NLRP3 interacts with the adaptor molecule apoptosis-associated speck-like protein (ASC) and pro-Caspase-1 to form the inflammasome [6]. Activated NLRP3 inflammasome promotes the proteolytic processing of pro-Caspase-1 into its active form, Caspase-1 (p20), and then cleaves pro-interleukin (IL)-1β and pro-IL-18 to mature bioactive forms IL-1β and IL-18, respectively. Two signals are required for the NLRP3 inflammasome formation and activation. One of these signals is the danger-associated molecular pattern (DAMP) including lipopolysaccharide (LPS), LPS primes the expression of NLRP3, pro-IL-1β, and pro-IL-18. The other stimulus, such as adenosine 5′-triphosphate (ATP), activates the NLRP3 inflammasome [7]. Hence, LPS and ATP was used in combination as NLRP3 inflammasome activator in our study.

NLRP3 inflammasome is an emerging key player in the development and progression of cancers, but its role in tumorigenesis and stimulating antitumor immunity are complex. Recent evidence showed that overexpressed and constitutively activated NLRP3 inflammasome contributed to the progression of human melanoma cells [8], lung cancer cells [9], and colon cancer cells [10]. Moreover, activated NLRP3 inflammasome restrained the antitumor efficacy and promoted tumor growth when gemcitabine and 5-fluorouracil were used for cancer cell treatment [11]. However, the expression of NLRP3 inflammasome was downregulated in human hepatocellular carcinoma [12]. NLRP3 inflammasome functioned as a negative regulator of tumorigenesis during colitis-associated cancer [13]. IL-18 production downstream of the NLRP3 inflammasome is critically involved in the protection against colorectal tumorigenesis [14]. But the exact role of NLRP3 inflammasome in SCCHN remains unclear. Cancer stem cells (CSCs) play major roles in cancer initiation and progression, moreover, it may be a critical factor of metastasis in colorectal cancer [15]. Chronic inflammation is recently shown to possibly regulate and enhance the development and function of CSCs [16]. IL-1β may promote epithelial mesenchymal transitions and stem cell development, as well as contribute to the malignancy, of colon cancer [17]. High IL-1β secretion is associated with malignant phenotype in the cancer microenvironment, and IL-1β may promote the inflammatory cycle in the cancer microenvironment that induces sterile inflammation and carcinogenesis [18]. Whether NLRP3 inflammasome has beneficial or detrimental effect on the carcinogenesis and development of cancer stem cells in SCCHN remains unknown.

In this study, we investigated the roles of NLRP3 inflammasome and CSCs in SCCHN. We initially assessed the expression of NLRP3 inflammasome and CSCs markers BMI1, ALDH1 and CD44 in SCCHN tissues and analyzed the correlation between NLRP3 inflammasome and CSCs markers. Then we investigated the potential roles of NLRP3 inflammasome in tumor carcinogenesis and self-renewal capacity of cancer cells in SCCHN cell lines and transgenic mouse SCCHN model.

## Methods

### Cell culture and reagents

SCCHN cell lines CAL27, SCC9, SCC25, and FaDu were purchased from the American Type Culture Collection and cultured according to the manufacturer’s online instructions. The immortalized oral keratinocyte line HIOEC from primary normal human oral epithelial cells infected with HPV16E6E7 (established in the Ninth People’s Hospital, Shanghai Jiao Tong University School of Medicine) was cultured in a defined keratinocyte serum-free medium (KSFM; GIBCO BRL, USA) [19]. LPS, ATP and MCC950 (PZ0280) were purchased from Sigma-Aldrich and the treatment of LPS, ATP and MCC950 were carried out as previously described [20]. The following antibodies were used in this study: Rabbit Polyclonal anti- NLRP3 (Atlas Antibodies AB, Sweden), Goat Polyclonal anti- ASC (GeneTex Inc., US); Rabbit Polyclonal anti- Caspase-1, Rabbit Monoclonal anti- IL-1β (Cell Signaling Technology, US); Rabbit Monoclonal anti- IL-18 (Abcam, UK); Rabbit Polyclonal anti- ALDH1 and Rabbit Polyclonal anti- BMI1 (GeneTex Inc., US); Mouse Monoclonal anti- CD44 (Cell Signaling Technology, US); Rabbit monoclonal anti- CD44(epitomics, UK).

### Tissue microarrays

The SCCHN tissue microarrays of humans used in this study were obtained from January 2008 and August 2014 in the Department of Oral and Maxillofacial Surgery, School and Hospital of Stomatology Wuhan University. The clinical stages of their SCCHN were classified according to the guidelines of the International Union Against Cancer (UICC 2002), and histological grading was determined according to the scheme of the World Health Organisation. Custom made tissue arrays of formalin-fixed tissues from SCCHN mentioned above were constructed with 1.5 mm core. These tissue microarray (T12–412-1 and T12–412-2) slides included 64 confirmed cases of SCCHN, 38 normal oral mucosa and 12 oral epithelial dysplasia. All slides were scanned at 400 magnification using an Aperio CS Scanscope (Aperio, CA) and quantified using the available Aperio algorithms. Written approval was obtained from all patients before the initiation of this study. All protocols dealing with the patients conformed to the ethical guidelines of the Helsinki Declaration and were approved by the Medical Ethics Committee of Hospital of Stomatology Wuhan University.

### Spontaneous SCCHN mouse models and MCC950 in vivo treatment

Time inducible tissue specific *Tgfbr1/Pten* 2cKO mice (*K14-Cre* ^ERtam^; *Tgfbr1* ^flox/flox^; *Pten* ^flox/flox^), *Tgfbr1* cKO mice (*K14-Cre* ^ERtam^; *Tgfbr1* ^flox/flox^), *Pten* cKO mice (K14-Cre^ERtam^; *Pten* ^flox/flox^) were maintained and genotyped according to published protocols [21]. The tamoxifen treatment procedure has been previously described [21]. *Tgfbr1* and *Pten* knockout mice were fully penetrated and developed oral and head neck carcinoma in 3–6 weeks. *Tgfbr1*/*Pten* 2cKO mice were baseline induced with 2 mg of tamoxifen for five consecutive days to delete *Tgfbr1* and *Pten*. 14 days after gavage, 10 mg/kg MCC950 was administered intraperitoneally every day for the first 3 days and every other day for 20 consecutive days (Fig. 3a). Tumor growth was assessed every day after tamoxifen gavage. All the mice were maintained in FVBN/CD1/129/C57 mixed background. All animal studies were approved and supervised by the Animal Care and Use Committee of Wuhan University, which were carried out according to the Institutional Guidelines for the use of laboratory animals in specific pathogen free Animal Laboratory of Wuhan University School and Hospital of Stomatology.

### Inflammasome activation and ELISA assays

For NLRP3 inflammasome stimulation, cells were primed with LPS for 6 h (10 ng/mL, Sigma-Aldrich). Then medium was replaced with serum free medium (SFM) containing DMSO (1:1000), MCC950 (10, 25, 50 nM) for 1 h, and subsequently incubated with adenosine ATP for 1 h (5 nM, Sigma-Aldrich) before collecting the supernatants [20, 22]. The concentrations of IL-1β in the culture supernatants were analyzed using commercially available IL-1β Enzyme-linked immunosorbent assay (ELISA, Dakewe BioTech, China) kits according to the manufacturer’s instructions.

### Sphere-forming assay and colony formation assay

For the sphere-forming assay, single-cell suspensions were resuspended in culture media with 1% N2 supplement (Gibco), 10 ng/mL bFGF (Invitrogen), and 10 ng/mL EGF (Gibco) and plated in ultra-low attachment plates as our previously reported [23]. Irnverted microscope and 96 well plate (Corning, US) were used to count the tumor sphere number. For colony formation assay, single-cell suspensions were seeded in 6 well plate (NEST Biotechnology Co. LTD.) at a density of 500 cells/well with 10% FBS contained-medium. After 7 days incubation, the colonies were fixed with 4% paraformaldehyde and stained by crystal violet. The numbers of colonies were counted. Each assay was performed in triplicate.

### Immunohistochemistry

Tumors from *Tgfbr1/Pten* 2cKO mice were dissected and fixed as previously described [21], and slides were stained with the appropriate antibody using a standard immunohistochemical staining protocol as previously described [24, 25]. The immunohistochemical staining was scanned using an Aperio ScanScope CS whole slice scanner (Vista, CA, USA) with background subtraction. The positive result was quantified using Aperio Quantification software for membrane, nuclear, or pixel quantification and histoscore were calculated using formula (3+) × 3 + (2+) × 2 + (1+) × 1 as previous described [26].

### Cell immunofluorescence

Cells were seeded on a cover glass slide chamber (Millipore, USA). After fixing with 4% paraformaldehyde at room temperature for 15 min, cells were treated with 0.5% triton X-100 and blocked with 2.5% BSA at room temperature for 1 h, and then incubated with primary antibody mentioned above overnight at 4 °C. Cells were then incubated with secondary fluorescent antibodies (DyLight 488 anti-rabbit, DyLight 594 anti-rabbit; Thermo Scientific, USA) with DAPI (Jackson ImmunoResearch Laboratories, Inc., West Grove, PA) for 1 h in the dark at room temperature. The slides were observed by a confocal laser scanning microscope (FV300, Olympus Life Science).

### Western blotting

The Western blotting analysis was conducted as previously described [27]. Briefly, cultured cells, tumors and normal mucosa from the buccal mucosa and tongue were collected from mice, then the protein lysates were generated using M-PER or RIPA reagent (Pierce, Rockford, IL) containing a complete mini protease inhibitor cocktail and phosphate inhibitors (Roche, Branchburg, NJ). After denaturation the total protein was separated using 10% SDS–polyacrylamide gel electrophoresis and transferred onto polyvinylidene fluoride membranes (Millipore). The blots were then blocked with 5% non-fat dry milk at room temperature for 1 h, and incubated overnight with the corresponding primary antibodies at dilutions recommended by the suppliers at 4 °C, finally by incubation with horseradish peroxidase-conjugated secondary antibody (Pierce, Rockford, IL). Next, the blots were detected using an enhanced chemiluminescence detection kit (West Pico, Thermo). GAPDH was detected on the same membrane and used as a loading control.

### Statistical analysis

Statistical data analysis was performed with GraphPad Prism 6 (GraphPad Software, Inc., La Jolla, CA) statistical packages. We analyzed the data between 2 experimental groups using unpaired *t* test and between multiple groups using a one-way ANOVA test. Overall survival curves were estimated by the Kaplan–Meier method and compared by the log-rank test. All data were presented as mean ± SEM, statistical significance was defined as the *p*-value was <0.05. *P < 0.05; ** *P* < 0.01; *** *P* < 0.001; ns, not significant.

## Results

### Increased NLRP3 inflammasome was associated with CSCs markers in human SCCHN specimen

**Fig. 1:**
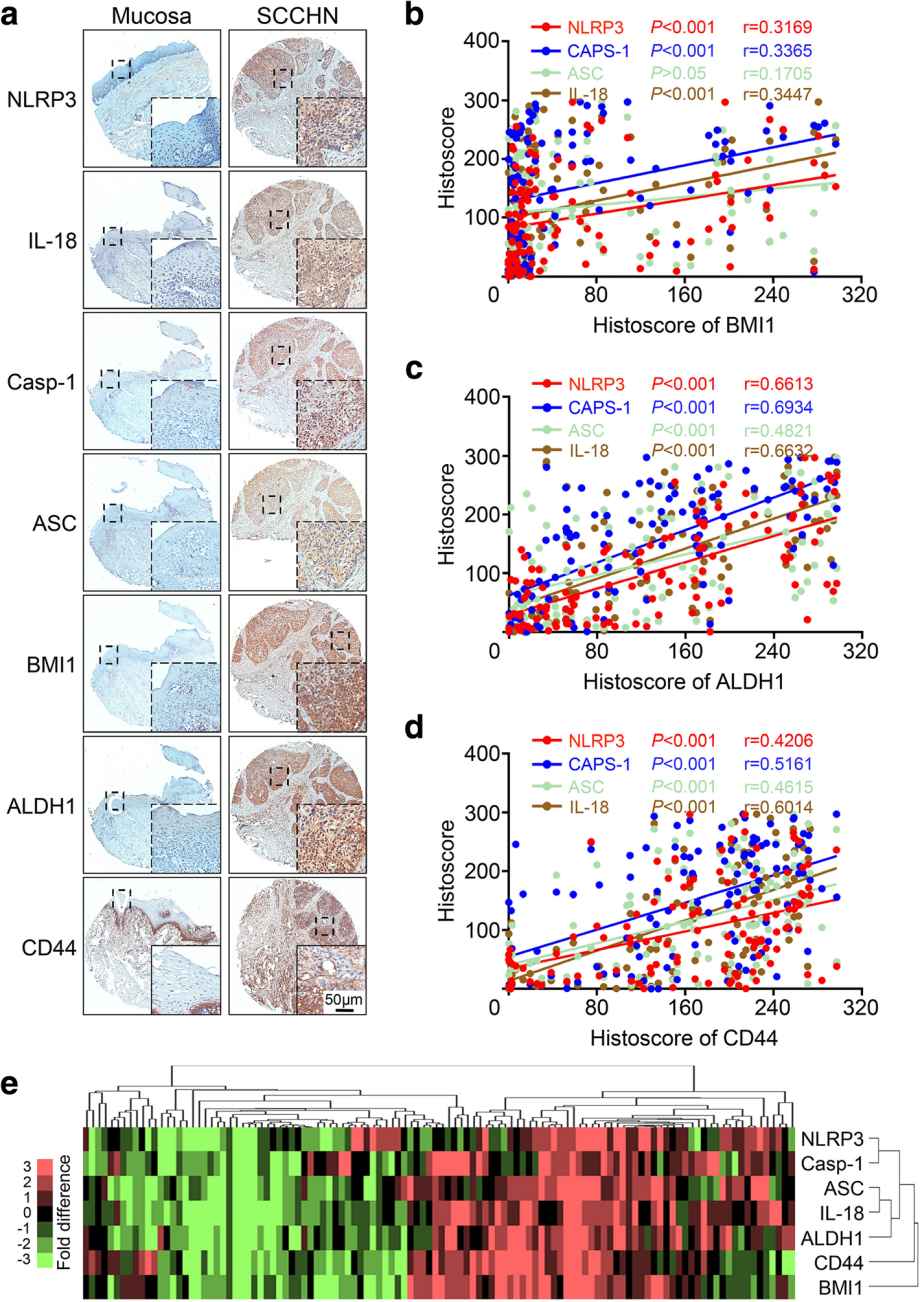
Increased NLRP3 inflammasome is correlated with CSCs markers BMI1, ALDH1 and CD44 in human SCCHN. **a** Representative Immunohistochemistry staining of NLRP3 inflammasome components (NLRP3, Caspase-1, ASC, and IL-18) and cancer stem cell markers BMI1, ALDH1 and CD44 in oral mucosa (left) and in human SCCHN tissue (right). Scale bar = 50 μm. **b** BMI1 expression was statistically associated with NLRP3 (*P* < 0.001, *r* = 0.3169), Caspase-1(*P* < 0.001, *r* = 0.3365), and IL-18 (*P* < 0.001, *r* = 0.3447) but not with ASC (*P* > 0.05, *r* = 0.1705), **c** ALDH1 expression was statistically associated with NLRP3 (*P* < 0.001, *r* = 0.6613), Caspase-1 (*P* < 0.001, *r* = 0.6934), ASC (*P* < 0.001, *r* = 0.4821) and IL-18 (*P* < 0.001, *r* = 0.6632), and **d** CD44 was statistically associated with NLRP3 (*P* < 0.001, *r* = 0.4206), Caspase-1 (*P* < 0.001, *r* = 0.5161), ASC (*P* < 0.001, *r* = 0.4615) and IL-18 (*P* < 0.001, *r* = 0.6014) by Pearson’s correlation coefficient test. **e** Hierarchical clustering of NLRP3, Caspase-1, ASC, IL-18, BMI1, ALDH1, and CD44. Immunohistochemical staining was clustered with cluster and visualized with Java Treeview

To determine whether NLRP3 inflamasome was associated with human HNSCC, we examined their expression by immunohistochemistry and found that NLRP3, Caspase-1, ASC, IL-18 were highly expressed in the cytoplasm of cancer cells, and the expression of them was negative or low in the normal mucosa (Fig. 1a). BMI1, ALDH1 and CD44 have been proposed as putative markers for the identification and isolation of CSCs in head and neck cancer [28, 29]. Our results showed that the protein expression of ALDH1 was positively stained in the cytoplasm, whereas BMI1 was in the cytoplasm and nucleus in SCCHN (Fig. 1a); CD44 was mainly expressed in the membrane of cancer cells but also in the basal layer of normal mucosa (Fig. 1a). These data indicated that BMI1, ALDH1 and CD44 were upregulated in the SCCHN tissues, but the correlation between NLRP3 inflammasome and CSCs remains unclear.

Increasing body of evidence from studies on various tumor types showed that chronic inflammation may regulate CSCs and enhance the development and function of the cancer cells [30]. However, the relationship between NLRP3 inflammasome and CSCs has not been investigated in human SCCHN. Interestingly, that data showed that the expression of BMI1 was statistically associated with NLRP3 (*P* < 0.001, *r* = 0.3169), Caspase-1 (*P* < 0.001, *r* = 0.3365) and IL-18 (*P* < 0.001, *r* = 0.3447) but not with ASC (*P* > 0.05, *r* = 0.1705); ALDH1 was statistically associated with NLRP3 (*P* < 0.001, *r* = 0.6613), Caspase-1 (*P* < 0.001, *r* = 0.6934), ASC (*P* < 0.001, *r* = 0.4821) and IL-18 (*P* < 0.001, *r* = 0.6632), and CD44 was statistically associated with NLRP3 (*P* < 0.001, *r* = 0.4206), Caspase-1 (*P* < 0.001, *r* = 0.5161), ASC (*P* < 0.001, *r* = 0.4615) and IL-18 (*P* < 0.001, *r* = 0.6014) as shown by the results of the Pearson correlation coefficient test (Fig. 1b-d). Results from hierarchy clustering and linear regression analyses showed that the expression of tumor-associated ASC and IL-18 are closer to ALDH1 expression, NLRP3 and Caspase-1 are close to ALDH1 expression, and NLRP3, Caspase-1, ASC, and IL-18 are close to CD44 and BMI1 expression (Fig. 1e).

### Increased NLRP3 inflammasome and CSCs markers in *Tgfbr1/Pten* 2cKO mouse SCCHN model

**Fig. 2:**
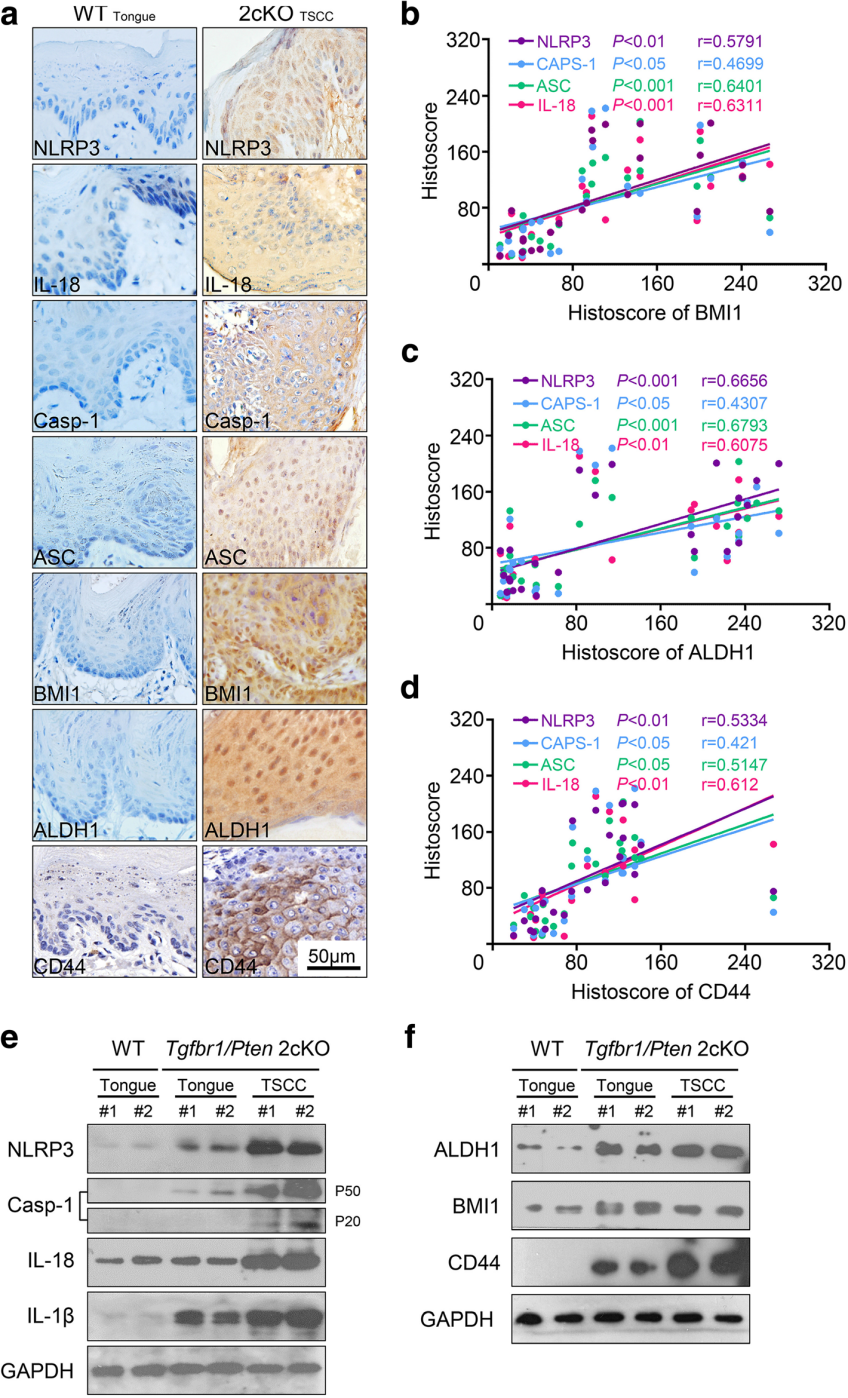
Increased expression of NLRP3 inflammasome and CSCs maker BMI1, ALDH1 and CD44 in *Tgfbr1*/*Pten* 2cKO mice. **a** Representative IHC staining images of NLRP3, Caspase-1, ASC, IL-18, ALDH1, and BMI1 in wild-type (WT) mice tongue and *Tgfbr1*/*Pten* 2cKO mice tumor (TSCC). Scale bars = 50 μm. (**b**, **c** and **d**) Pearson’s correlation coefficient test of BMI1 expression with NLRP3 (*P* < 0.01, *r* = 0.5791), Caspase-1(*P* < 0.05, *r* = 0.4699), ASC (*P* < 0.001, *r* = 0.6401) and IL-18 (*P* < 0.001, *r* = 0.6311); ALDH1 expression was statistically related to NLRP3 (*P* < 0.001, *r* = 0.6656), Caspase-1 (*P* < 0.05, *r* = 0.4307), ASC (*P* < 0.001, *r* = 0.6793) and IL-18 (*P* < 0.01, *r* = 0.6075) and CD44 expression was statistically related to NLRP3 (*P* < 0.01, *r* = 0.5334); Caspase-1 (*P* < 0.05, *r* = 0.421); ASC (*P* < 0.05, *r* = 0.5147); IL-18 (*P* < 0.01, *r* = 0.612) in 12 wild type mice tongues and 12 mice SCCHN. (**e** and **f**) Western blot analysis showed a significant increase in NLRP3, Caspase-1, IL-1β, IL-18, BMI1, ALDH1 and CD44 in *Tgfbr1*/*Pten* 2cKO mice than in the control group

NLRP3 inflammasome is a key player in the progression of cancers, but its role in tumorigenesis and tumor environment are complex. A spontaneous de novo SCCHN mice model for tumorigenesis studies was applied to further determine the potential role of NLRP3 inflammasome in tumor initiation effect. First we immunostained the proteins of the NLRP3 inflammasome components to investigate whether NLRP3 inflammasome was activated in *Tgfbr1/Pten* 2cKO mice. Our results revealed intense staining of NLRP3, ASC, Caspase-1, and IL-18 expression in *Tgfbr1/Pten* 2cKO SCCHN mice tumor lysates compared with control wild type mice tongues (Fig. 2a), these results indicated that the NLRP3 inflammasome was activated, and very high level of NLRP3 inflammasome was found in *Tgfbr1/Pten* 2cKO SCCHN mouse model. Moreover, we found the expression of BMI1, ALDH1 and CD44 was also upregulated in the *Tgfbr1/Pten* 2cKO SCCHN mice tumor (Fig. 2a). Additionally, the Pearson correlation coefficient test results shown that BMI1 expression was closely related to NLRP3 inflammasome in SCCHN mice model (NLRP3, *P* < 0.01, *r* = 0.5791; Caspase-1, *P* < 0.05, *r* = 0.4699; ASC, *P* < 0.001, *r* = 0.6401; IL-18 *P* < 0.001, *r* = 0.6311; Fig. 2b), ALDH1 was the same (NLRP3, *P* < 0.01, *r* = 0.6656; Caspase-1, *P* < 0.05, *r* = 0.4307; ASC, *P* < 0.001, *r* = 0.6793; IL-18 *P* < 0.01, *r* = 0.6075; Fig. 2c); and CD44 was also correlated with NLRP3 inflammasome (NLRP3, *P* < 0.01, *r* = 0.5334; Caspase-1, *P* < 0.05, *r* = 0.421; ASC, *P* < 0.05, *r* = 0.5147; IL-18 *P* < 0.01, *r* = 0.612; Fig. 2d). In addition, our western blot results showed that NLRP3, Caspase-1, IL-18 and IL-1β were highly expressed in the *Tgfbr1*/*Pten* 2cKO mice SCCHN tumor lysates compared with control wild type tongues, and the expression of BMI1, ALDH1 and CD44 were consistent with NLRP3 inflammasome (Fig. 2e and f).

### NLRP3 inflammasome blockade by MCC950 delayed tumorigenesis in *Tgfbr1/Pten* 2cKO mouse SCCHN model

**Fig. 3:**
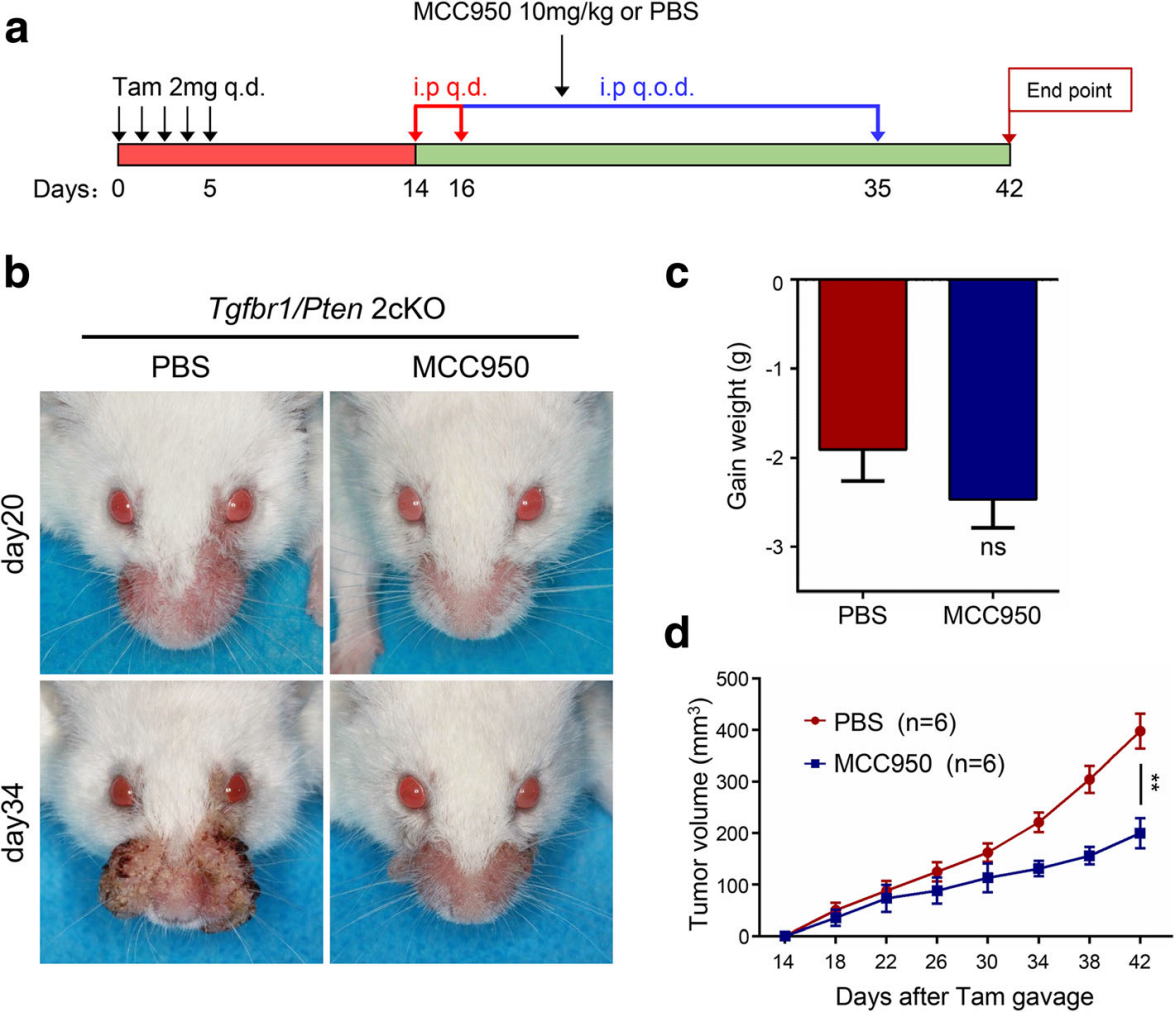
Treatment of MCC950 in *Tgfbr1/Pten* 2cKO mice SCCHN. **a** Schematic diagram represents the MCC950 delivery strategy in *Tgfbr1/Pten* 2cKO mice. Oral administration of tamoxifen was conducted consecutively for 5 days. Mice received 10 mg/kg MCC950 or control PBS 100 μl through intraperitoneal injection every day for the first three days and every other day for 20 consecutive days. Data are presented as mean ± SEM, *n =* 6. **b** Representative photos show that head and neck tumorigenesis was delayed after MCC950 treatment compared with the control group. **c** Gain weight to the mouse sacrifice deadline of MCC950 and control-treated mice. ns, not significant. **d** Tumor volume curve showed that MCC950 treatment delayed the growth of head and neck tumor (**P < 0.01)

We previously reported that the loss of *Tgfbr1* and *Pten* results in cellular senescence evasion, cancer-related inflammation, and expansion of the CSCs in the basilar epithelial layer in SCCHN of the *Tgfbr1/Pten* 2cKO mice. The inflammation-related marker STAT3, CXCL1 [31, 32], and CSCs-related markers CD44, CD133, Sox2, and Oct4 were overexpressed within *Tgfbr1/Pten* 2cKO mouse SCCHN model [32–34]. We determined whether MCC950 treatment could modulate the inflammation environment in 2cKO SCCHN model. A specific small molecule inhibitor of NLRP3 inflammasome MCC950 was applied (Fig. 3a). We found that MCC950 treatment (*n =* 6) significantly reduced tumor burden of the head–neck (Fig. 3b) compared with control group (*n =* 6). Moreover, MCC950 treatment caused no weight loss compared with the PBS treatment group (Fig. 3c). In addition, the tumor growth curve of the head and neck demonstrated that MCC950 treatment effectively reduced the tumor burden (Fig. 3d).

### NLRP3 inflammasome blockade by MCC950 decreased CSCs phenotype in *2cKO* mouse SCCHN model

**Fig. 4:**
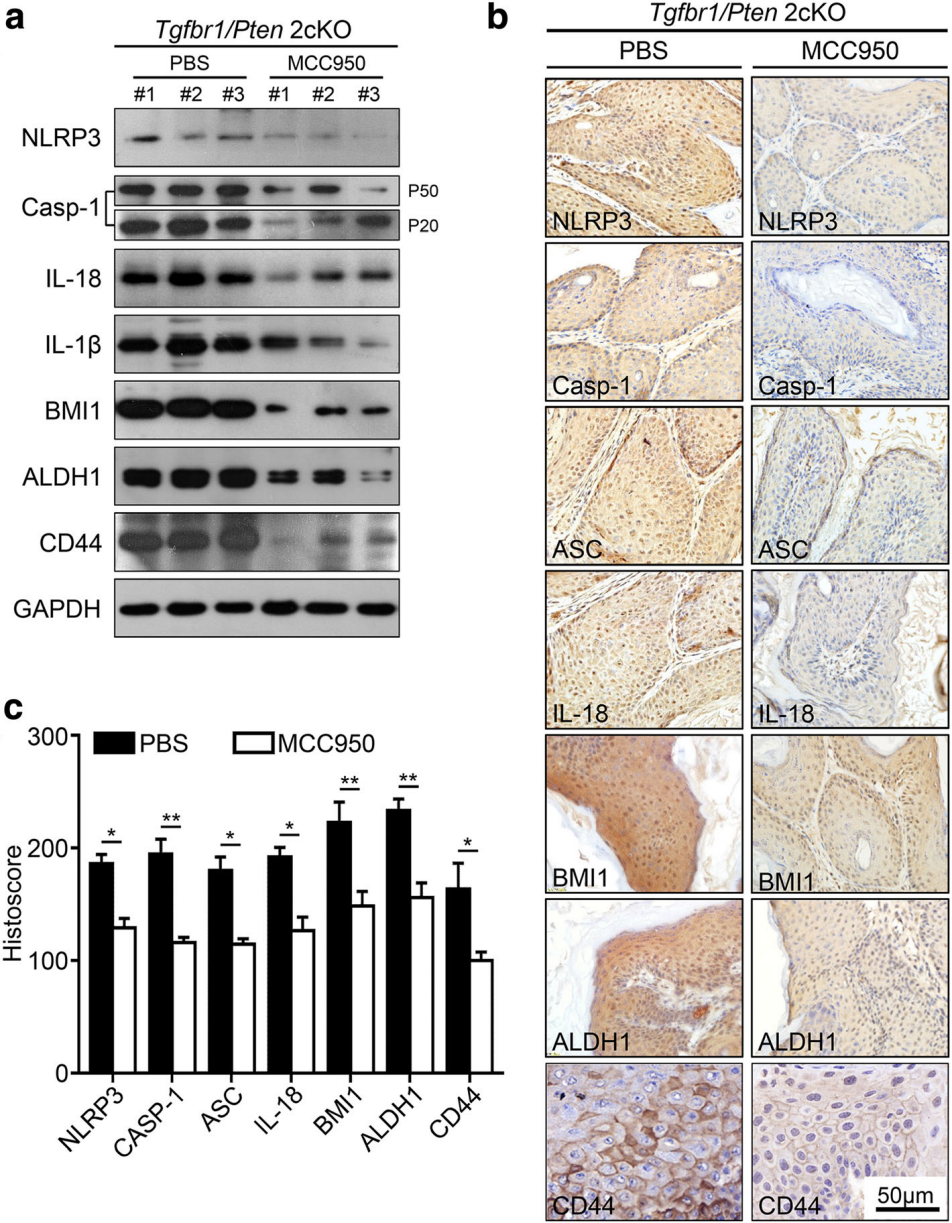
MCC950 inhibits NLRP3 inflammasome-induced CSCs formation in *Tgfbr1/Pten* 2cKO mice. **a** Western blot analysis showed that the expression of NLRP3 inflammasome and CSCs makers BMI1, ALDH1 and CD44 were downregulated in the MCC950 treatment group than in the control group. **b** Immunohistochemical staining of NLRP3 inflammasome, BMI1, ALDH1 and CD44 in *Tgfbr1/Pten* conditional knockout mice after MCC950 treatment. Scale bars = 50 μm. **c** Quantitative analysis showed that the expression of NLRP3 inflammasome, BMI1, ALDH1 and CD44 was significantly reduced in the MCC950 treatment group than in the control group (Mean ± SEM, **P < 0.01; *P < 0.05)

To evaluated the potential role of inflammation on CSCs markers during SCCHN progression and the use of the novel anti-inflammatory NLRP3 inflammasome inhibitor MCC950 arising de novo in 2cKO mouse SCCHN model. Our results from the Western blot analysis showed that the expression of NLRP3, Caspase-1, IL-1β, IL-18, BMI1, ALDH1 and CD44 was also significantly downregulated by MCC950 treatment (Fig. 4a). Moreover, the immunohistochemical staining analysis showed that MCC950 treatment remarkably reduced the expression of NLRP3, Caspase-1, ASC, IL-18, BMI1, ALDH1 and CD44 (*P < 0.05; **P < 0.01, Fig. 4b and c) compared with the control group. Therefore, NLRP3 inflammasome blockade could delay tumor initiation and progression by modulating the inflammation environment and regulating the population of CSCs in *Tgfbr1/Pten* 2cKO mouse SCCHN model.

### NLRP3 inflammasome could be activated by LPS and ATP stimulation in SCCHN cell lines

**Fig. 5:**
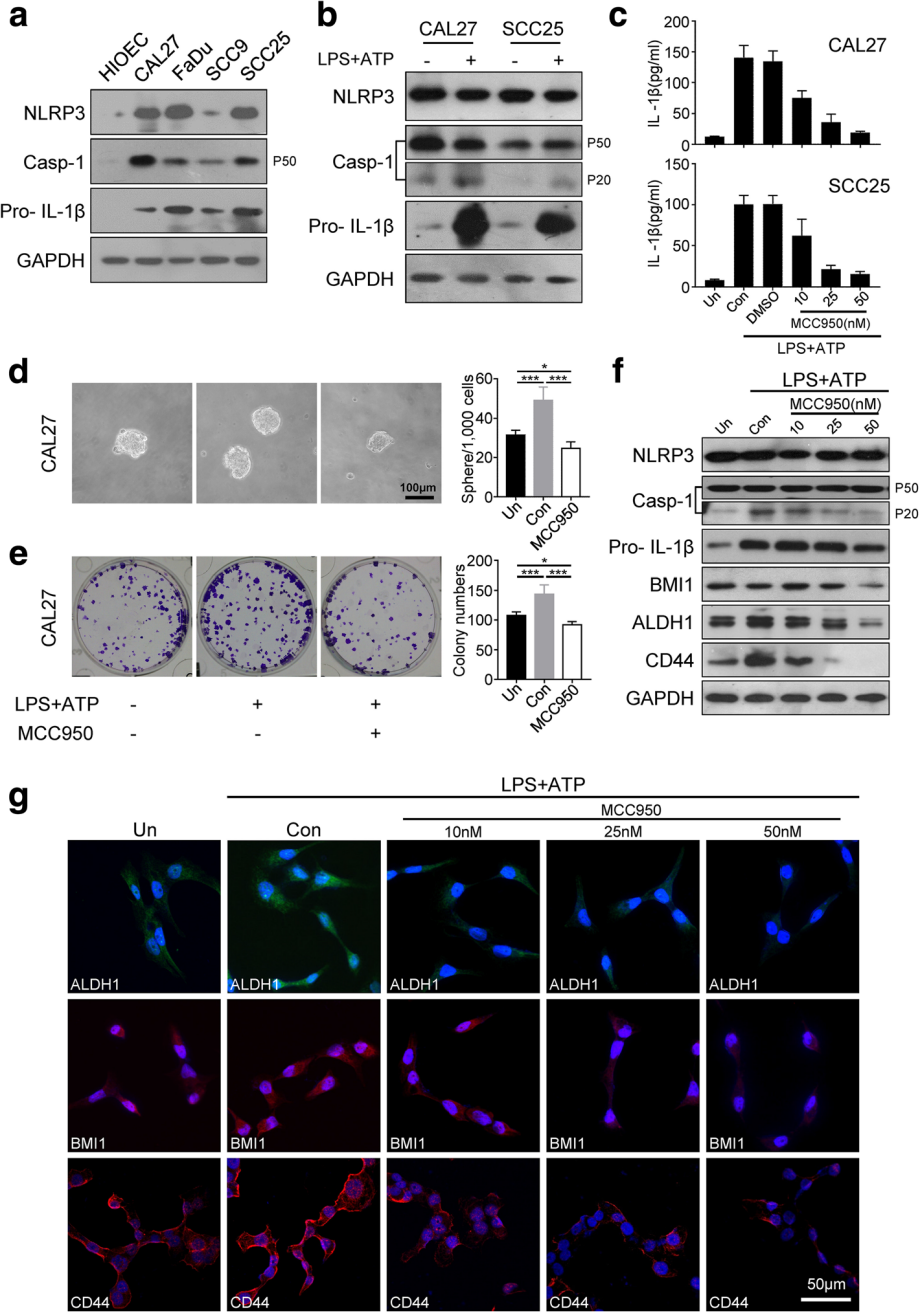
NLRP3 inflammasome regulates CSCs formation in SCCHN cell lines. **a** Western blot analysis of NLRP3 inflammasome in SCCNH cell line and human immortalized oral epithelial cell lysates for comparison. **b** Caspase-1(P20) and Pro- IL-1β in CAL27 and SCC25 cells were significantly increased after stimulation with LPS(10μg/ml, 6 h) and ATP(5 nM, 1 h) as shown by Western blot analysis. **c** Production of IL-1β (mature form) from CAL27 and SCC25 cells stimulated with LPS and ATP and treated with MCC950(1 h) as measured by ELISA. Data are expressed as mean ± SEM of three independent experiments performed in triplicate. (**d** and **e**) LPS and ATP significantly induced CAL27 cells sphere and colony formation compared with the control group, and MCC950 significantly reduced spheres and colonies; Scale bar = 100 μm. (Mean ± SEM, ***P < 0.001; *P < 0.05). **f** The expression of NLRP3 inflammasome, BMI1, ALDH1 and CD44 was analyzed by Western blot after treatment with LPS + ATP(6 h) and MCC950(12 h) in CAL27. **g** CAL27 cells were treated with LPS + ATP(6 h) and 10, 25, and 50 nM MCC950(12 h). The cells were analyzed by immunofluorescence cytochemistry. ALDH1 (green), BMI1 and CD44 (red), DAPI (blue); Scale bar = 50 μm. Data shown are representative of three experiments

Cumulative studies demonstrated that inflammasomes, including NLRP3, could be activated by microbial pathogens and cancer [20]. Two signals are required for the NLRP3 inflammasome formation and activation. One of these signals is the danger-associated molecular pattern (DAMP) including lipopolysaccharide (LPS), LPS primes the expression of NLRP3, pro-IL-1β, and pro-IL-18. The other stimulus, such as adenosine 5′-triphosphate (ATP), activates the NLRP3 inflammasome [7]. Hence, LPS and ATP was used in combination as NLRP3 inflammasome activator in our study. We detected stronger levels of NLRP3, Caspase-1, pro-IL-1β, and IL-18 in CAL27, FaDu, SCC9, and SCC25 than in the control cell (Fig. 5a). Hence, we selected CAL27 and SCC25 cell lines with high expression of NLRP3, Caspase-1, and pro-IL-1β for the following in vitro functional assay. As we can see, the level of both pro-IL-1β and IL-1β were obviously elevated after the stimulation of LPS and ATP in the CAL27 and SCC25 cell lines as shown by the results from ELISA and Western blot analysis (Fig. 5b and c). Thus, NLRP3 inflammasome was obviously activated by LPS and ATP stimulation.

### NLRP3 inflammasome blockade by MCC950 affected the population of CSCs in SCCHN cell lines

Sphere-forming assay and colony formation assay were performed to investigate the potential role of NLRP3 inflammasome in the self-renewal capacity of cancer cells. MCC950 was used to further investigate whether CSCs could be reduced by blockade of NLRP3 inflammasome in SCCHN cell lines. Contrary to other NLRP3 inflammasome inhibitors, MCC950 blocks ASC oligomerization to inhibit canonical and non-canonical NLRP3 inflammasome activation rather than by reducing NLRP3 protein expression [35]. First, the level of IL-1β was obviously downregulated after stimulation with LPS and ATP and treatment with MCC950 at 10, 25, and 50 nM by ELISA in the CAL27 and SCC25 cell lines (Fig. 5c). LPS- and ATP-treated CAL27 cells were found to form more spheres and colonies than the control, which indicates LPS and ATP possible promotion of self-renewal of SCCHN cells in vitro (Fig. 5d and e). MCC950-treated group after LPS and ATP stimulation significantly showed lower number of spheres and colonies than in the LPS- and ATP-treated groups (Fig. 5d and e). BMI1, ALDH1, CD44 and the expression of NLRP3 inflammasome were also analyzed by Western blot to further confirm the effect of MCC950 on stemness. The results show that the expression of NLRP3, pro-Caspase-1(P50), and pro-IL-1β were not obviously downregulated by MCC950 after LPS and ATP treatment, because MCC950 treatment could alter the expression of IL-1β and activated form of Caspase-1(P20) but not that of NLRP3, pro-IL-1β [20]. Additionally, BMI1, ALDH1 and CD44 expression was significantly downregulated after MCC950 treatment (Fig. 5f). we performed immunofluorescence to further verify whether the inhibition of self-renewal capacity by MCC950 was through those CSCs related makers. The positive expression of BMI1, ALDH1 and CD44 was significantly upregulated by LPS and ATP treatment but downregulated by MCC950 treatment in dose-dependent compared with the control group (Fig. 5g). Thus, NLRP3 inflammasome activation could promote self-renewal capacity along with upregulated expression of stemness markers. Moreover, NLRP3 inflammasome blockade could reduce self-renewal capacity concomitant with downregulated expression of stemness markers in SCCHN cell lines.

## Discussion

Chronic inflammation is an important event in carcinogenesis and tumor progression, and cancer-related inflammation has been identified as the seventh hallmark of cancer [36]. Cancer stem cell is viewed as the seed of cancer, recently chronic inflammation is shown to possibly regulate and enhance the development and function of CSCs [16]. NLRP3 inflammasome plays a key role in inflammation, and recent evidence showed that activation of NLRP3 inflammasome was correlated with carcinogenesis and progression in cancers. However, the roles of NLRP3 inflammasome in different cancers are cell- and tissue-specific [8, 13]. SCCHN is an inflammation-related cancer [37], and the expression and function of NLRP3 inflammasome have not been clarified in SCCHN. In the present study, we demonstrated that NLRP3 inflammasome components were overexpressed in human SCCHN tissues and *Tgfbr1/Pten* 2cKO mouse SCCHN model. Moreover, NLRP3 inflammasome was correlated with CSCs markers BMI1, ALDH1 and CD44. NLRP3 inflammasome activation could promote CSCs formation, and NLRP3 inflammasome blockade could reduce CSCs formation in SCCHN cell lines and *Tgfbr1/Pten* 2cKO mouse SCCHN model. These results suggest that NLRP3 inflammasome might play potential roles on CSCs regulation in human SCCHN.

NLRP3, a functional component of the inflammasome, with ASC and Caspase-1, regulates the maturation of IL-1β and IL-18 in immune cells. A previous study showed that NLRP3 was upregulated in prostate cancer cells, and hypoxia could contribute to prostatic chronic inflammation and activate the NLRP3 inflammasome [38]. NLRP3 inflammasome components NLRP3, ASC, and Caspase-1 were found to be highly expressed in lung cancer cell lines and tissues than in adjacent normal tissues [22]. Our results were consistent with these studies, but the novel finding in our study was that the expression of NLRP3 significantly upregulated in SCCHN tissues and cancer cell lines. Nevertheless, NLRP3 inflammasome was demonstrated as a negative regulator of tumorigenesis in colitis-associated and liver cancers [12, 13]. These findings indicate that NLRP3 inflammasome in different cancers is cell- and tissue-specific. Moreover, NLRP3 inflammasome components ASC and Caspase-1 were also highly expressed in SCCHN tissues and cancer cell lines. ASC has been associated with epithelial skin carcinogenesis [39], and ASC-mediated inflammation signaling pathway played an important role in the intestinal tumorigenesis and inflammasome-mediated IL-1β secretion of mice [40]. IL-1β is the most extensively investigated inflammasome-related cytokine in cancers. This molecule is significantly increased in many kinds of cancers, such as gastric, prostate, and tongue carcinomas [37, 41, 42]. NLRP3 inflammasome components IL-1β and IL-18 were found to be highly expressed in lung cancer cell lines and tissues than in cancer-adjacent normal tissues [22]. These results suggest that the level of NLRP3 inflammasome play an important role in the carcinogenesis and CSCs phenotype of SCCHN.

Interestingly, NLRP3 inflammasome can be activated in a sterile setting by necrotic cancer cells [43]. Chronic over activation of the IL-1β pathway has been considered as a tumor promoting condition, which is beneficial for IL-1β inhibition for tumor prevention or therapy. IL-1β was correlated with poor prognosis [44]. In this report, the expression of ASC was found to be correlated with overall survival in SCCHN (Additional file 1: Fig. S1). A recent study has demonstrated that high level of ASC expression was correlated with poor overall survival, disease specific survival, and disease-free survival in oral cavity squamous cell carcinoma [45]. Moreover, inactivation of NLRP3 inflammasome by MCC950 could not reduce NLRP3, pro-Caspase-1 protein expression level in vitro, but we observed decreased expression of NLRP3 and pro-Caspase-1 in the MCC950 treated *Tgfbr1/Pten* 2cKO SCCHN mice model compared to control group. Because when the tumor occurs, the NLRP3 inflammasome was activated and produced IL-1β to form an inflammatory microenvironment and promoted tumor progression, and the progressive tumors would further stimulate NLRP3 inflammasome to produce more IL-1β. Given that blockade of NLRP3 inflammasome could delay tumorigenesis in SCCHN mice by reducing the production of IL-1β, and the stimulation on NLRP3 inflammasome from early tumor is less than advanced tumor, which might account for the decreased expression of NLRP3 and pro-Caspase-1. These results reiterate that NLRP3 inflammasome-mediated inflammation contributes to the development and progression of SCCHN.

CSCs are perceived to initiate and sustain tumor growth, pathways involved in inflammation have been reported to enhance CSCs populations [17]. Our results showed that the cancer expression of stem cell markers BMI1, ALDH1 and CD44 were highly upregulated in human SCCHN and mice SCCHN tissues, and NLRP3 inflammasome was closely associated with those makers. Our observation indicates that inflammation may regulate CSCs in SCCHN. In breast cancer, wound healing and inflammation enhanced CSCs populations, leading to local and systemic recurrences after treatment [30]. In colon cancer, IL-1β promoted epithelial mesenchymal transitions and stem cell development [17]. In pancreatic cancer, a preclinical proof of concept had been identified to target the inflammation initiation to inhibit CSCs early to improve the treatment of pancreatic cancers [46]. Notably, previous studies indicated that IL-1β might be a new therapeutic target against CSCs in colon cancer [17] and oral squamous cell carcinoma [47]. Current treatments for NLRP3-related diseases include biologic agents that target IL-1β [20]. Our results showed that NLRP3 inflammasome activation by LPS and ATP stimuli could promote sphere-forming capacity along with upregulated expression of BMI1, ALDH1 and CD44. Moreover, NLRP3 inflammasome blockade by MCC950 could reduce self-renewal capacity along with downregulated expression of BMI1, ALDH1 and CD44 in SCCHN cell lines. Furthermore, MCC950 treatment modulated the NLRP3 inflammasome-induced inflammation environment and regulated the population of CSCs in *Tgfbr1/Pten* 2cKO mouse SCCHN model. These observations indicated that the NLRP3 inflammasome might play a key role in CSCs regulation and may be a novel therapeutic target in SCCHN.

## Conclusions

In summary, inflammation is closely correlated with carcinogenesis in head and neck squamous cell carcinoma. In this report, we identified that NLRP3 inflammasome was upregulated and associated with carcinogenesis and CSCs self-renewal activation. Additionally, MCC950 treatment blocked NLRP3 inflammasome and regulated the population of CSCs in *Tgfbr1/Pten* 2cKO mouse SCCHN model. Hence, NLRP3 inflammasome can be a potential target aimed at CSCs in the development of novel approaches for head and neck cancer therapy.

## Additional file

Additional file 1: Fig. S1. The relationship between NLRP3 inflammasome and overall survival. Kaplan-Meier survival curve of ASC indicated high ASC expression level suggest poorer prognosis (*P* < 0.05). (TIFF 1283 kb)

## Abbreviations

ATP: adenosine 5′-triphosphate
CSCs: cancer stem cells
DAMP: danger-associated molecular pattern
ELISA: Enzyme-linked immunosorbent assay
LPS: lipopolysaccharide
SCCHN: squamous cell carcinoma of the head and neck
