# Supplementary figures and images for: NLRP3 inflammasome activation promotes inflammation-induced carcinogenesis in head and neck squamous cell carcinoma
Source: J Exp Clin Cancer Res. 2017 Sep 2;36:116. doi: 10.1186/s13046-017-0589-y (PMC5581464; doi:10.1186/s13046-017-0589-y)

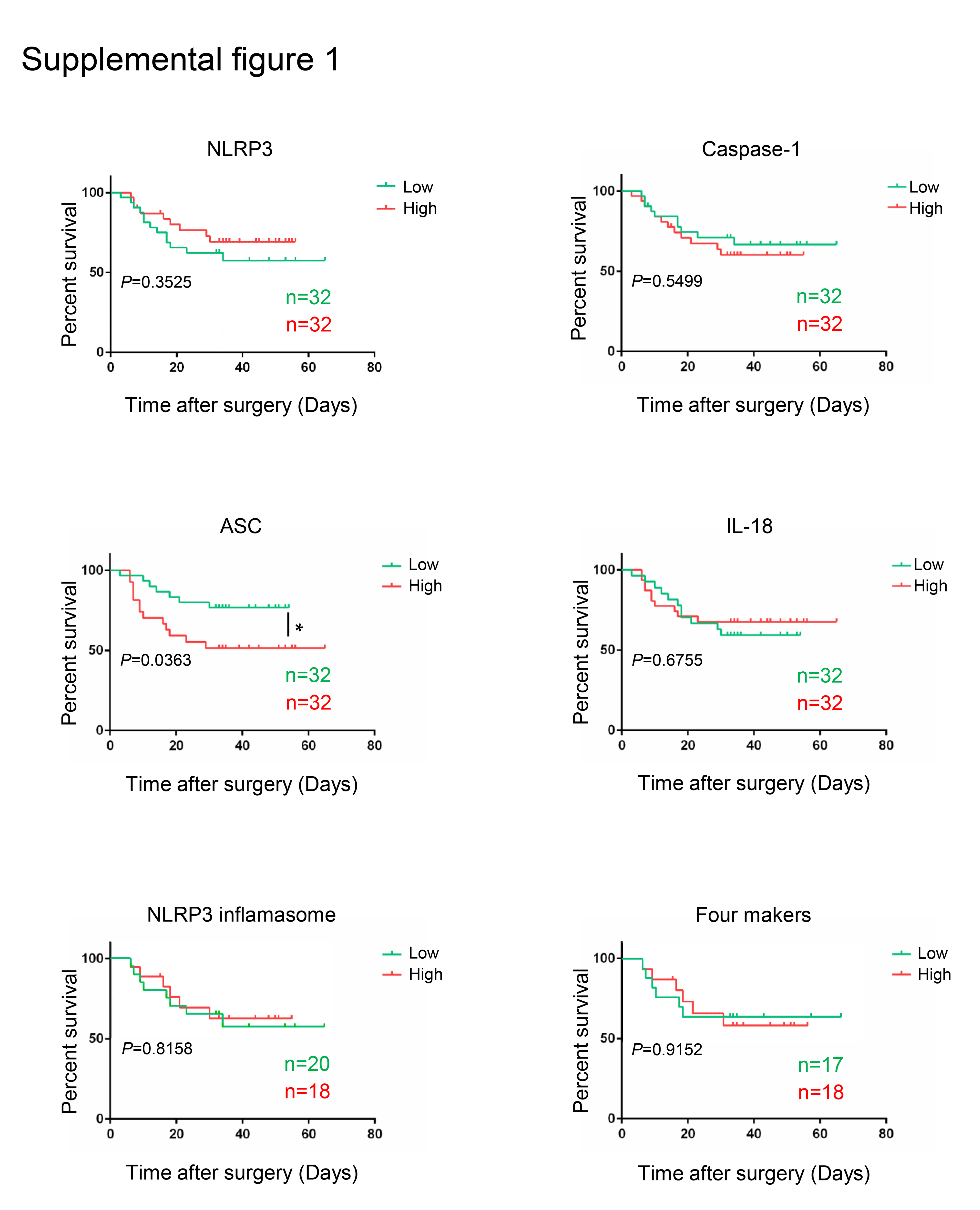

Supplement: Additional file 1: Fig. S1. — The relationship between NLRP3 inflammasome and overall survival. Kaplan-Meier survival curve of ASC indicated high ASC expression level suggest poorer prognosis (P < 0.05). (TIFF 1283 kb) [file 13046_2017_589_MOESM1_ESM.tif]
